# Rice of Northeast India harbor rich genetic diversity as measured by SSR markers and Zn/Fe content

**DOI:** 10.1186/s12863-019-0780-6

**Published:** 2019-10-23

**Authors:** Vanlalsanga, S. Priyokumar Singh, Y. Tunginba Singh

**Affiliations:** Department of Botany, Mizoram University, Aizawl, Mizoram India

**Keywords:** Northeast India, Rice landraces, Genetic diversity, Microsatellite marker, Zn/ Fe content

## Abstract

**Background:**

Rice (*Oryza sativa* L.) is one of the most important crops of the world and a major staple food for half of the World’s human population. The Northeastern (NE) region of India lies in the Indo-Burma biodiversity hotspot and about 45% of the total flora of the country is found in the region. Local rice cultivars from different states of NE India were analyzed for genetic diversity and population structure using microsatellite markers, and their zinc and iron content.

**Results:**

A total of 149 bands were detected using twenty-two microsatellite markers comprising both random and trait-linked markers, showing 100% polymorphism and high value of expected heterozygosity (0.6311) and the polymorphism information content (0.5895). Nali Dhan cultivar of Arunachal Pradesh possessed the highest genetic diversity (0.3545) among studied populations while Moirangphou Khonganbi of Manipur exhibited the lowest genetic diversity (0.0343). The model-based population structure revealed that all the studied 65 rice cultivars were grouped into two clusters. Cluster I was represented by 36 cultivars and cluster II by 29 cultivars. Badalsali cultivar of Assam possessed the highest Zn content (75.8 μg/g) and Kapongla from Manipur possessed the lowest (17.98 μg/g). The highest and the lowest Fe content was found in Fazu (215.62 μg/g) and Idaw (11.42 μg/g) of Mizoram.

**Conclusion:**

The result suggested rice cultivars of NE India possessing high genetic diversity (Nali dhan), high Zn (Badalsali) and Fe (Fazu) content can be useful as a source of germplasm for future rice improvement programs.

## Background

Rice (*Oryza sativa* L.) is the staple food for more than 50 % of the world’s population. The rice production and consumption in Asia alone accounts for more than 90 % of the global rice yields [1]. It is imperative to develop measures to improve global rice production to warrant food security for increasing human populations. Although rice production has increased to about two folds in the past few decades with the introduction of improved varieties and proper crop management strategies, the need for high yielding, better varieties still remain unchanged. Bouis and Welch [2] suggested that increased rice productivity and the ability to deliver all the essential nutrients is crucial to meet both the energy needs and adequate nutritional health for the people in developing countries. Kennedy et al. [3] have reported that more than two billion people are affected with Iron (Fe), Iodine (I), Zinc (Zn), and vitamin A deficiencies, especially in poor families of developing countries, of which more than five million children die every year due to nutrient malnutrition [2]. Fe and Zn are essential micronutrients for all forms of life due to their functional importance in cell development and gene expression [4, 5]. Zn deficiency is known to be one of the most important malnutrition problems [6]. The effects of Zn deficiency include growth retardation, diarrhea, emotional disorders, reduction or absence of hormone secretion in male adolescents, rough skin, poor appetite, mental lethargy, delayed wound healing, weight loss, etc. [7]. Fe deficiency leads to blood loss, mal-absorption, chronic diseases, genetic disorders, etc. [8–11]. Increased Zn and Fe uptakes are required during crucial metabolic periods such as early human growth and pregnancy, so children and pregnant women are at higher risk of these nutrients deficiency [6, 12, 13]. It has been suggested by rice workers that the development of rice varieties with higher nutrient content may improve the nutritional health of people whose major diet is rice.

The Northeastern states of India, comprising Arunachal Pradesh, Assam, Manipur, Meghalaya, Mizoram, Nagaland, and Tripura, lies within the international boundaries of Bhutan and China in the north, Bangladesh in the southeast and Myanmar in the west. This region constitutes the Indo-Burma biodiversity hotspot [14] and is inhabited by various ethnic groups of people who speak different dialects and perform different cultural practices. The topography and biogeography of the region make this place a picturesque and also rich in biodiversity of flora and fauna. About 45% of the total flora of the country is found in the region [15]. This region harbors the richest genetic diversity reservoir for agri-horticultural crops. Rice cultivation provides the main source of food and employment for the people of this region as most of the population is involved in agriculture and allied activities. About 72% of the total cultivated area is under agricultural cultivation practices in upland, lowland, and water fed areas [15]. Although a large number of rice cultivars are available, most of the rice cultivated in the region are high yielding varieties (HYV) developed using modern genetic engineering tools. This trend implies a possible narrowing of the natural gene pool. However, it is also surprising to know that the many indigenous farmers of the hilly areas are still practicing their own landrace or cultivar cultivation that they inherit from their forefathers, which suit the local microclimate and adaptation. The cultural importance of the local landraces is also depicted by these people.

Knowledge on the extent of genetic variation and relationship among genotypes is necessary for developing more effective breeding and conservation programs [16], Understanding and utilizing the genetic diversity in crop plants is crucial for sustaining the increasing global and local food demands [17]. Assessment of the genetic diversity of local rice landraces or cultivars will provide a valuable source as it can be useful for crop improvement programs, Integrated Pest Management (IPM) measures and sustainable development of agriculture. Rice varieties of this region possess unique traits which are of great interest to the plant breeders. Some of the useful qualities identified in these landraces include unique adaptive traits for cold tolerance, flooding and salt tolerance, etc. [15]. Many molecular markers have been used to assess genetic diversity within and between populations. Among them, microsatellite or SSR (Simple sequence repeat) are one of the most preferred for assessment of genetic diversity because they are reliable, rapid, easy to score, cost-effective and require only a small amount of DNA [18, 19]. The present study was performed to assess the genetic diversity of the local rice landraces of the Northeast states of India using SSR markers, with two aims i) to estimate the Zn and Fe contents and ii) to facilitate conservation and utilization of these landraces.

## Results

### Zn and Fe content

Table 1 summarizes the Zn and Fe content of rice cultivars used in this study. Zn content in the studied cultivars ranged from 17.98 μg/g to 75.8 μg/g with an average of 36.65 μg/g (Table 1). Badalsali cultivar of Assam possessed the highest Zn content and Kapongla of Manipur possessed the lowest. The Zn contents of Northeast rice cultivars (38.55 μg/g) were higher than that of improved varieties (32.17 μg/g) used in the current investigation.

**Table 1:**
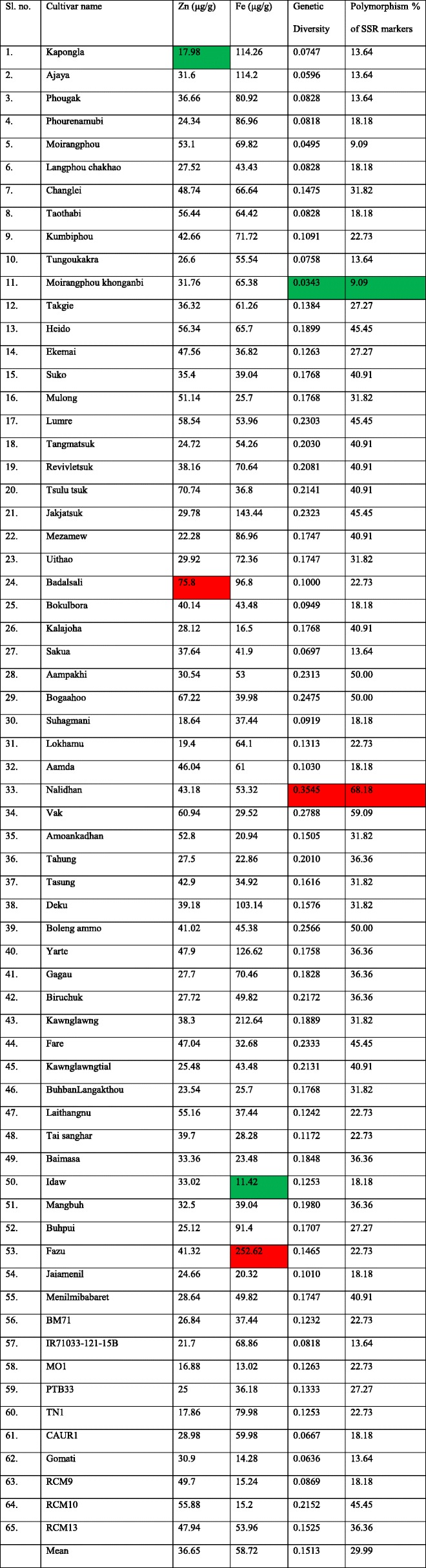
Zn and Fe content, Gene diversity and percentage polymorphism in the studied cultivars

Highest value cell was indicated in red colour and lowest value cell was indicated in green colour for each parameter

Fe content ranged from Idaw (11.42 μg/g) to Fazu (215.62 μg/g) with an average of 59.29 μg/g (Table 1). Similar to Zn, Fe contents of Northeast rice cultivars (62.9 μg/g) were higher than that of improved varieties (39.41 μg/g) in the present study.

### SSR polymorphism

**Fig. 1:**
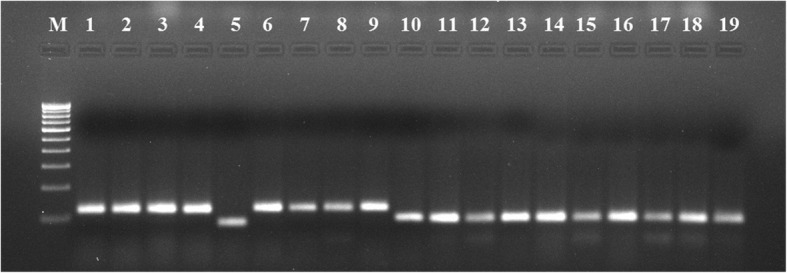
A 2.5% agarose gel showing the banding pattern of Assam rice cultivars generated by RM1. M represents a 100 bp DNA ladder. Lane 1–9 Suhagmani, 10–19 Lokhamu

**Fig. 2:**
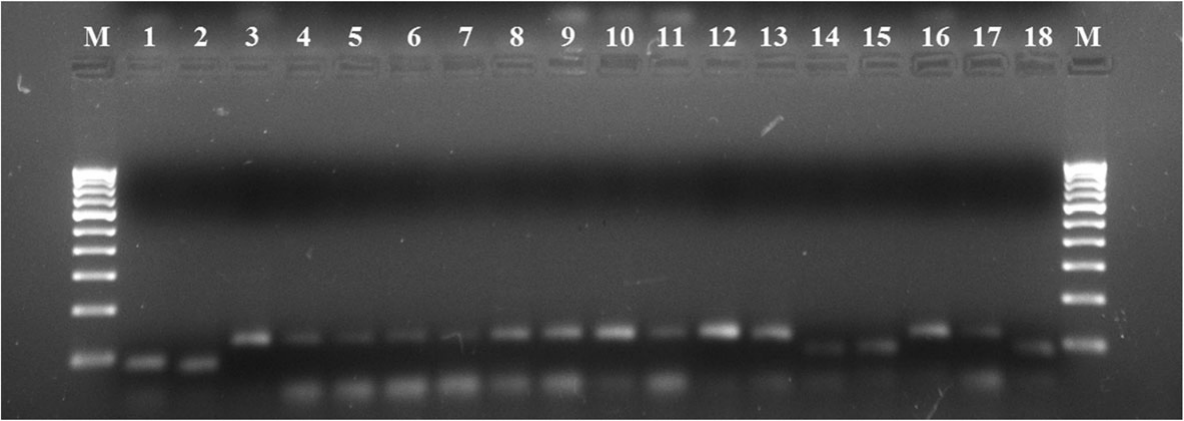
A 2.5% agarose gel showing the banding pattern of Nagaland rice cultivars generated by RM1. M represents a 100 bp DNA ladder. Lane 1–2 Heido, 3–12 Ekemai, 13–18 Suko

The agarose gels showing banding patterns of some rice cultivars were presented in Figs. 1 and 2. Table 2 shows a summary of the genetic markers used in the current study. A total of 149 bands were detected using twenty-two SSR primers. All twenty-two SSR markers were found to be polymorphic (100% polymorphism). The average number of alleles per locus was 6.7727 and the maximum number of band [12] was generated by RM223 and the minimum [2] was generated by RM315. The mean number of effective alleles was found to be 3.3080. Major allele frequency (MAF) ranged from 0.2431 (RM246) to 0.9723 (RM443) with an average of 0.4973. Expected heterozygosity (H_E_) varied from 0.1157 (RM315) to 0.8466 (RM72) with an average of 0.6311 respectively. Nei’s genetic diversity varied from 0.1155 (RM315) to 0.8453 (RM72) with an average of 0.6301. Fst ranged from 0.5739 (RM223) to 0.9619 (RM443) with an average of 0.7870. Polymorphism information content (PIC) ranged from 0.1012 (RM315) to 0.8274 (RM72) averaging 0.5895. The summary statistics of each marker is shown in Table 2.

**Table 2:** Summary of markers used in the present study

Locus	na	ne	MAF	H_E_	Nei	Fst	PIC
RM1	10.0000	4.2403	0.3662	0.7653	0.7642	0.8164	0.7359
RM154	8.0000	5.1918	0.3200	0.8086	0.8074	0.7367	0.7439
RM131	11.0000	2.5082	0.6123	0.6022	0.6013	0.5789	0.5826
RM135	9.0000	5.5756	0.2738	0.8219	0.8206	0.5358	0.7963
RM153	4.0000	1.9540	0.5815	0.4890	0.4882	0.9389	0.3726
RM125	5.0000	1.9837	0.6231	0.4967	0.4959	0.7735	0.4161
RM72	11.0000	6.4654	0.2569	0.8466	0.8453	0.7703	0.8274
RM171	5.0000	2.5417	0.5308	0.6075	0.6066	0.7768	0.5570
RM287	4.0000	2.7942	0.4846	0.6431	0.6421	0.7614	0.6151
RM302	5.0000	1.4456	0.8154	0.3087	0.3082	0.8473	0.2922
RM3825	4.0000	2.4690	0.5600	0.5959	0.5950	0.9255	0.5624
RM246	10.0000	5.6321	0.2431	0.8237	0.8224	0.7969	0.8036
RM260	6.0000	3.5529	0.3662	0.7196	0.7185	0.8784	0.6710
RM525	6.0000	2.8050	0.5431	0.6445	0.6435	0.9460	0.5986
RM219	5.0000	2.8788	0.4815	0.6536	0.6526	0.9071	0.5998
RM315	2.0000	1.1306	0.9292	0.1157	0.1155	0.8721	0.1012
RM223	12.0000	3.4552	0.4985	0.7117	0.7106	0.5739	0.7060
RM8094	8.0000	3.4626	0.3646	0.7123	0.7112	0.6881	0.6587
RM493	8.0000	3.9689	0.3431	0.7492	0.7480	0.8976	0.7055
RM3412	7.0000	3.9858	0.3231	0.7503	0.7490	0.9166	0.7209
RM443	3.0000	1.4768	0.9723	0.3234	0.3229	0.9619	0.2471
RM169	6.0000	3.2572	0.4523	0.6941	0.6930	0.6990	0.6552
Mean	6.7727	3.3080	0.4973	0.6311	0.6301	0.7870	0.5895
SD ±	2.8273	1.4428	0.2034	0.1863	0.1860	0.1256	0.1920

*na* Number of alleles, *ne* Effective number of alleles, *MAF* Major allele frequency, *H*_*E*_ Expected heterozygosity, *Nei* Nei’s genetic distance, *Fst* Genetic differentiation, *PIC* Polymorphism information content

### Population structure analysis

**Fig. 3:**
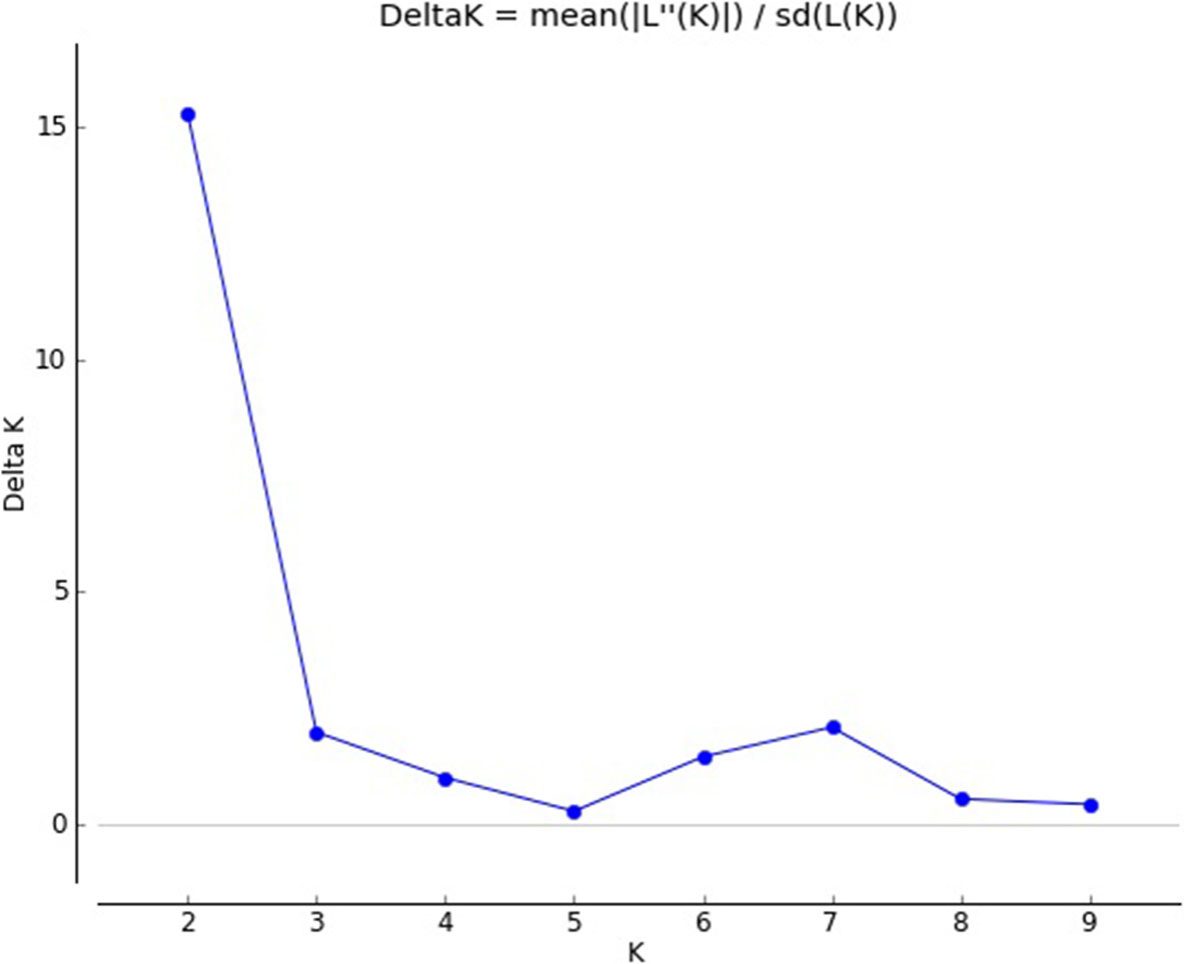
Relationship between ΔK and K showing a peak at K = 2

**Fig. 4:**
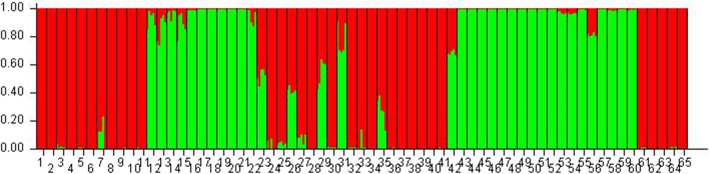
Population structure (barplot) of rice cultivars

**Fig. 5:**
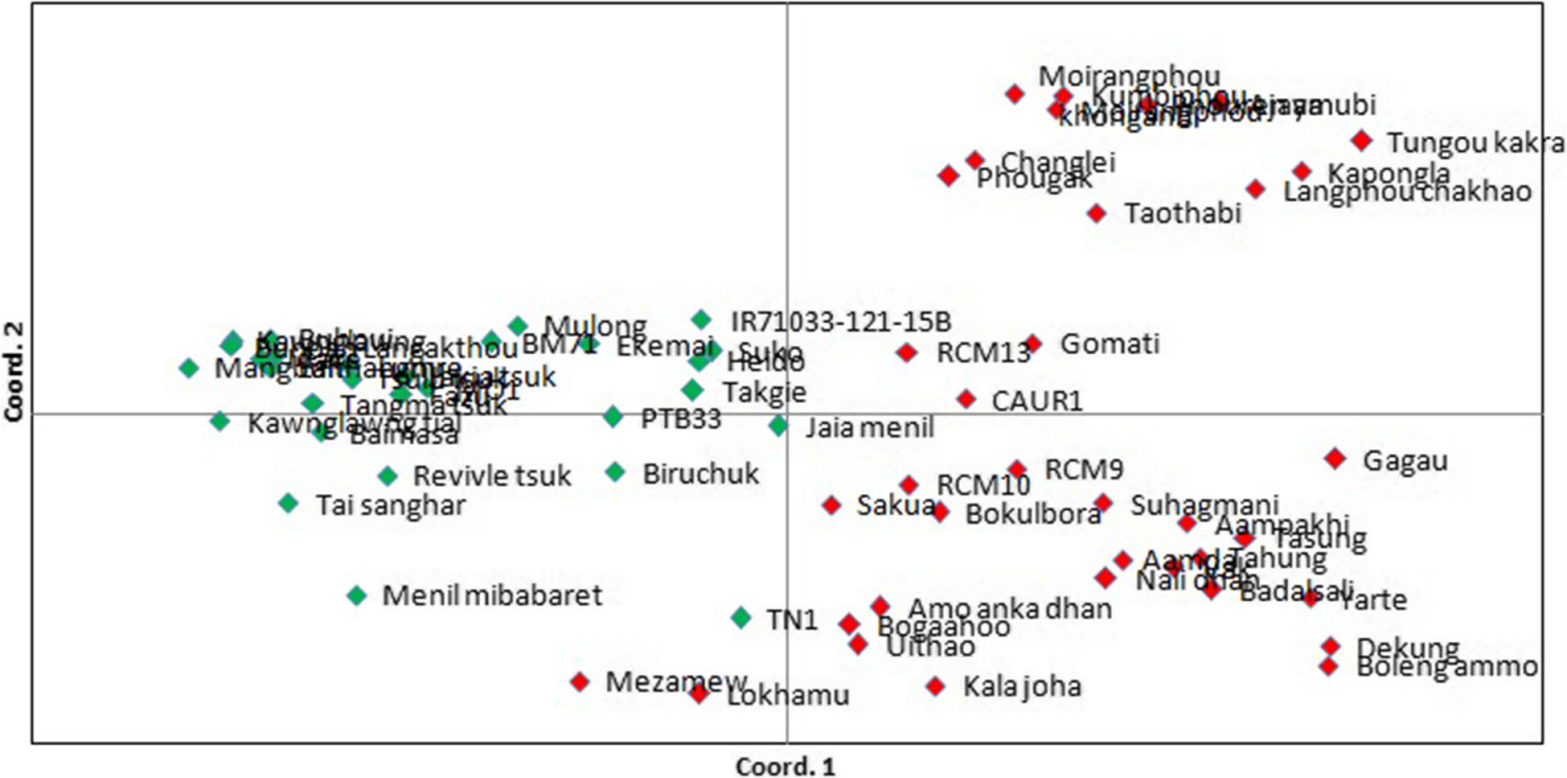
Principal coordinates analysis of 65 rice cultivars

**Fig. 6:**
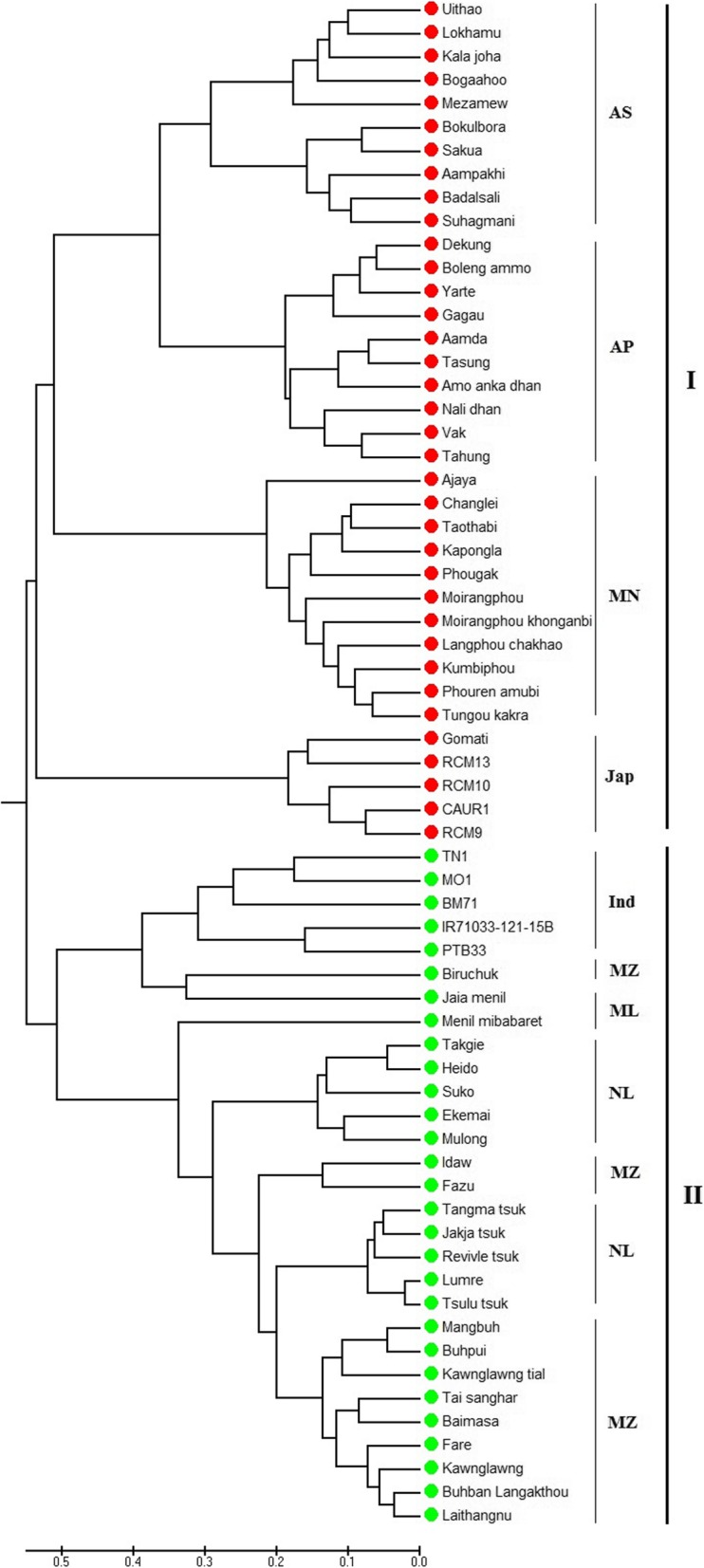
UPGMA tree based on Nei’s genetic distance (AS = Assam, AP = Arunachal Pradesh, MN = Manipur, MZ = Mizoram, ML = Meghalaya, NL = Nagaland, Ind = indica, Jap = japonica)

The model-based population structure analysis using STRUCTURE showed that the highest value of ΔK was at K = 2 (Fig. 3), grouping all the studied 65 rice cultivars into two clusters (Fig. 4), designated here as cluster I and cluster II. Principal Coordinates Analysis (Fig. 5) performed using GenAlEx and UPGMA tree (Fig. 6) constructed using MEGAfurther supplemented the STRUCTURE results. Both PCoA and UPGMA tree divided 65 rice cultivars into two groups. Cluster I was represented by 36 cultivars and cluster II was represented by 29 cultivars. In UPGMA tree, Cluster I was subdivided into four groups exhibiting rice cultivars of Manipur, Assam, Arunachal Pradesh, and *Japonica* varieties. And cluster II could also be subdivided into four groups comprising rice cultivars of Mizoram, Nagaland, Meghalaya and *Indica* varieties. Analysis of molecular variance (AMOVA) showed that the genetic variation of two clusters of 65 rice cultivars was distributed into 73% among populations and 27% within populations. Average distances (expected heterozygosity) between individuals in the same cluster varied from Cluster I (0.5197) to cluster II (0.5686). Fst values of Cluster I and Cluster II were found to be 0.2635 and 0.2107 respectively with an average of 0.2371. The mean alpha value was found to be 0.0663.

Population-wise diversity comparison (Table 1) showed that Nalidhan cultivar of Arunachal Pradesh possessed the highest genetic diversity (0.3545) among studied populations while Moirangphoukhonganbi of Manipur exhibited the lowest genetic diversity (0.0343). In state-wise comparison, the genetic diversity (H_E_) of Manipur rice cultivars ranged from 0.0343 (Moirangphoukhonganbi) to 0.1475 (Changlei), and that of Nagaland ranged from 0.1263 (Ekemai) to 0.2323 (Jakjatsuk), and Assam, from 0.0697 (Sakua) to 0.2475 (Bogaahoo), Arunachal Pradesh, from 0.1030 (Aamda) to 0.3545 (Nalidhan), Mizoram, from 0.1172 (Tai sanghar) to 0.2333 (Fare) and Meghalaya rice cultivars ranged from 0.1010 (Jaiamenil) to 0.1747 (Menilmibabaret). Rice cultivars of Arunachal Pradesh, in overall exhibited highest gene diversity (0.2022), followed by Nagaland (0.1896), Mizoram (0.1746), Assam (0.1492), Meghalaya (0.1378) and then Manipur (0.0788). The average genetic diversity of all indigenous cultivars was found to be 0.1575 which was higher than that of *indica* (0.1180) and *japonica* (0.1170) varieties.

**Fig. 7:**
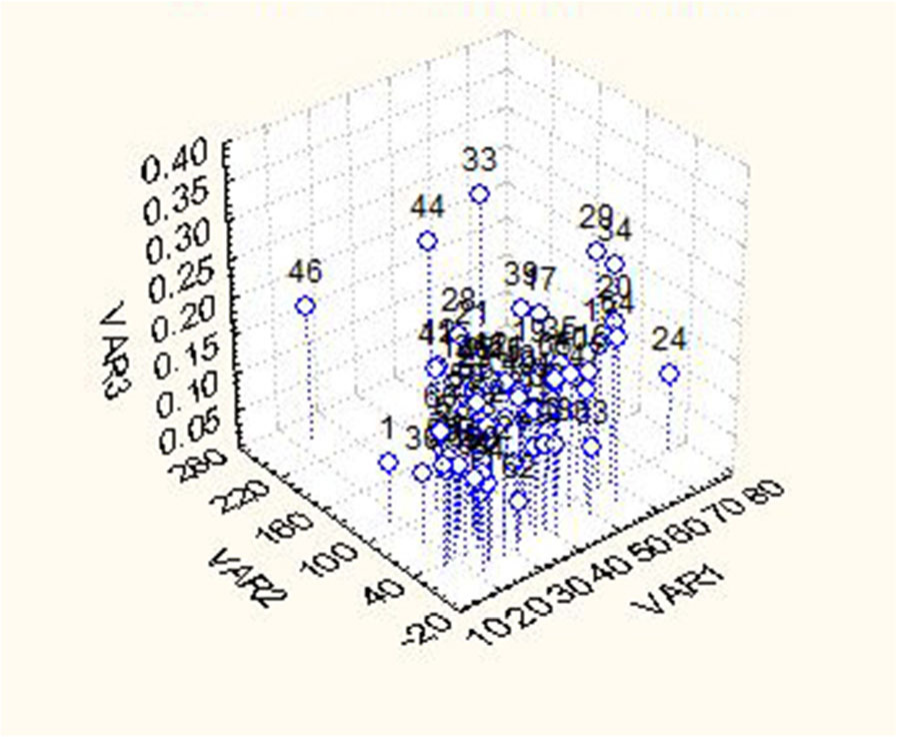
Relationship of SSR, Zn and Fe diversity (Var1 = Zn content, Var2 = Fe content, Var3 = SSR genetic diversity)

Comparative analysis of gene diversity, Zn and Fe content showed that there was no significant correlation among all the three parameters. However, few cultivars with high gene diversity also showed higher Zn and Fe content (Table 3, Fig. 7).

**Table 3:** Correlations of genetic diversity, Zn and Fe content of 65 rice varieties, indicating no significance for all the test entries at *p* < 0.05

	GD	Zn	Fe
GD	1		
	p = −--		
Zn	0.2265	1	
	*p* = .070	p = −--	
Fe	−0.0478	−0.032	1
	*p* = .705	*p* = .800	p = −--

*GD* Genetic diversity, *Zn* Zinc, *Fe* Iron

## Discussion

In this study, genetic structure and diversity analysis of 55 indigenous rice cultivars of Northeast India and 5 *indica* and 5 *japonica* test varieties were performed using twenty-two SSR markers comprising 9 random and 13 trait-linked markers, and Zn and Fe content. Assessments of genetic diversity of NE rice using molecular markers has been reported previously [20–25]. Though high genetic diversity was previously shown in the NE rice accessions, reports on micronutrients diversity are scarce. Micronutrient deficiencies to Zn and Fe, constitute the two most common nutrient deficiencies in humans [23, 26, 27], especially in developing countries [28]. Although rice is a major staple food for a large part of the world especially in Asia, it has been reported as a poor source of essential micronutrients and vitamins [29]. In the current study, relatively high Zn and Fe contents were detected in some of the cultivars. The Fe content in the present study was found to be higher than that of rice cultivars of West Bengal and adjoining areas, though zinc content was lower [23]. High Fe content was also previously reported in the Indian cultivars by Brar et al. [30]. The Zn content was higher and the Fe content was found to be lower than a previous report on local rice germplasm of Tripura state [31]. Average Zn and Fe contents in the present study were comparable with a previous report [30, 32]. In another report by Verma and Srivastav [33], among some aromatic and nonaromatic Indian rice cultivars, aromatic rice had higher Zn and Fe contents. Interestingly, Zn and Fe contents in the current study was found to be higher than the ones reported by Verma and Srivastav [33]. Therefore, to overcome the micronutrient deficiencies, the present study will be helpful for designing crop improvement programs, though more investigations are still needed to further find out higher contents of Zn and Fe since these micronutrients are essential for human health and development.

The NE rice cultivars contain considerable genetic diversity and variable traits which might be good sources for various improvement programs [20]. All SSR markers used in the present study were found to be polymorphic. A combination of random and trait-linked markers was utilized since Yadav et al. [34] reported trait-linked markers gave higher value of genetic diversity and Polymorphism Information Content (PIC) in some Indian rice germplasm than random markers, whereas several other workers have shown high genetic diversity in NE rice cultivars using random markers [20, 21, 24]. The number of alleles per locus (6.7727) found was higher than the ones reported earlier by Upadhyay et al. [35] (3.96 alleles per locus) and lower than that reported by Choudhury et al. [20] (13.57 alleles per locus). However, it was comparable with 7.9 alleles per locus reported by Das et al. [21]. The mean H_E_ and PIC found in the present study showed a high value of heterozygosity index. The mean Fst values for all loci and between the two clusters were found to be 0.7786 and 0.1987 respectively indicating very high genetic differentiation among loci and among the clusters. Based on SSR analysis, there were seventeen highly informative markers (PIC> 0.50), viz., RM1, RM154, RM131, RM135, RM72, RM171, RM287, RM3825, RM246, RM260, RM525, RM219, RM223, RM8094, RM493, RM3412 and RM169; two informative markers (PIC between 0.25 and 0.50), RM153, RM125, and RM302; and two slightly informative markers (PIC< 0.25), RM315 and RM443 [36, 37].

Population structure analysis using STRUCTURE showed highest ΔK value at K = 2 revealing that the studied 65 rice cultivars were grouped into two clusters. The number of the cluster was in agreement to the previous studies: two clusters among 29 varieties of cultivated rice of NE India [20] and two clusters among 6 landraces of North-Western Indian Himalayas [38]. Roy et al. [24] have also reported a similar result of K = 2, among hill rice of Arunachal Pradesh, NE India, belonging to *indica* and *japonica.* In the current study, the identified two main clusters can also be divided into sub-clusters corresponding to state-wise grouping. A similar result of state-wise grouping was also observed in aromatic rice germplasm from North Eastern India [13]. According to Evano et al. [39], alpha value closed to zero indicated that most of the individuals were from one population or another, and an alpha value greater than 1 indicated that most individuals were admixed. The observed small alpha value in this study (0.0663) might indicate that most of the individuals originated from one population or another.

In some areas of NE India, rice has been cultivated in shifting or jhum lands which only depend on the Monsoon rain. These cultivars survive in long spells of rainless weather and may be good candidates to look for these variable traits. Other important traits include dark color and aroma in *Chakhao* rice of Manipur, resistance against blast, resistance to gall midge, deep water tolerance in *Baon* of Assam, drought resistance in *Hmawrhang* of Mizoram, etc. [15, 40, 41]. As evident from the current study, the genetic diversity of indigenous rice cultivars was found to be higher than that of agronomically improved varieties. These results are in agreement to a similar pattern observed for rice varieties of the Eastern Himalayan region of Northeast India [20]. The use of such genetic variability in breeding programs is a key factor for crop improvement [42]. Among the studied rice cultivars, Nalidhan cultivar of Arunachal Pradesh possessed the highest genetic diversity, followed by Vak and Boleng ammo cultivars. These high genetic diversity cultivars are promising candidates as sources for effective breeding or future rice improvement programs. However, some cultivars such as Moirangphou khonganbi, Moirangphou possessed a low level of genetic diversity suggesting necessary actionsshould be taken on the conservation of these landraces. Cultivars such as Vak, Bogaahoo and Tsulu tsuk possessed high genetic diversity and high Zn concentration. Similarly, Kawnglawng, Jakjatsuk, Yarte, Mezamew, etc. possessed high Fe content and high genetic diversity. Nalidhan, the cultivar with the highest genetic diversity also possessed Zn and Fe contents higher than the average observed for these studied populations. Lumre also possessed high genetic diversity, high Zn and average Fe content. The highest Zn containing Badalsali cultivar possessed a lower genetic diversity than the average of all the studied populations. Similarly, the Fazu cultivar with the highest Fe content showed lower genetic diversity than the average of all the studied populations. The present investigation showed that the majority of the cultivars with high genetic diversity had high Zn contents and many cultivars also exhibited high genetic diversity along with high Fe content.

## Conclusion

The current study provides a better understanding of genetic structure, diversity, and micronutrient (Zn and Fe) richness in the indigenous rice cultivars of NE India. The cultivars possessing high genetic diversity (Nali dhan), high Zn (Badalsali) and Fe (Fazu) contents are promising candidates as parental lines for future rice breeding programs. These findings will further facilitate the conservation strategies and utilization of these landraces for developing sustainable rice improvement programs.

## Methods

### Plant material, collection and planting

Rice landraces were collected from six states of NE India. Details of collection sites are shown in Table 4. *Indica* and *japonica* check varieties were kind gifts from ICGEB, New Delhi, ABF, Hyderabad and ICAR, Kolasib. For isolation of DNA, individual cultivars were planted on polypots at Department of Botany, Mizoram University, India.

**Table 4:** Collected rice cultivars of NE India

Sl. No.	Cultivar name	Place of collection	States	Type
1.	Kapongla	Kakching	Manipur	Landrace
2.	Ajaya	Kakching	Manipur	Landrace
3.	Phougak	Kakching	Manipur	Landrace
4.	Phourenamubi	Thoubal	Manipur	Landrace
5.	Moirangphou	Thoubal	Manipur	Landrace
6.	Langphouchakhao	Kakching	Manipur	Landrace
7.	Changlei	Kakching	Manipur	Landrace
8.	Taothabi	Kakching	Manipur	Landrace
9.	Kumbiphou	Kakching	Manipur	Landrace
10.	Tungoukakra	Thoubal	Manipur	Landrace
11.	Moirangphoukhonganbi	Thoubal	Manipur	Landrace
12.	Takgie	Peren	Nagaland	Landrace
13.	Heido	Peren	Nagaland	Landrace
14.	Ekemai	Peren	Nagaland	Landrace
15.	Suko	Kohima	Nagaland	Landrace
16.	Mulong	Tuensang	Nagaland	Landrace
17.	Lumre	Tuensang	Nagaland	Landrace
18.	Tangmatsuk	Mokokchung	Nagaland	Landrace
19.	Revivletsuk	Mokokchung	Nagaland	Landrace
20.	Tsulu tsuk	Mokokchung	Nagaland	Landrace
21.	Jakjatsuk	Mokokchung	Nagaland	Landrace
22.	Mezamew	NC Hills	Assam	Landrace
23.	Uithao	NC Hills	Assam	Landrace
24.	Badalsali	Sonitpur	Assam	Landrace
25.	Bokulbora	Sonitpur	Assam	Landrace
26.	Kalajoha	Dheemaj	Assam	Landrace
27.	Sakua	Lakhimpur	Assam	Landrace
28.	Aampakhi	Lakhimpur	Assam	Landrace
29.	Bogaahoo	Dheemaj	Assam	Landrace
30.	Suhagmani	Dheemaj	Assam	Landrace
31.	Lokhamu	NC Hills	Assam	Landrace
32.	Aamda	West Siang	Arunachal Pradesh	Landrace
33.	Nalidhan	West Siang	Arunachal Pradesh	Landrace
34.	Vak	West Siang	Arunachal Pradesh	Landrace
35.	Amoankadhan	West Siang	Arunachal Pradesh	Landrace
36.	Tahung	East Siang	Arunachal Pradesh	Landrace
37.	Tasung	East Siang	Arunachal Pradesh	Landrace
38.	Dekung	West Siang	Arunachal Pradesh	Landrace
39.	Boleng ammo	East Siang	Arunachal Pradesh	Landrace
40.	Yarte	East Siang	Arunachal Pradesh	Landrace
41.	Gagau	East Siang	Arunachal Pradesh	Landrace
42.	Biruchuk	Lawngtlai	Mizoram	Landrace
43.	Kawnglawng	Diltlang South	Mizoram	Landrace
44.	Fare	Diltlang South	Mizoram	Landrace
45.	Kawnglawngtial	Mualbukawnpui	Mizoram	Landrace
46.	BuhbanLangakthou	Vawmbuk	Mizoram	Landrace
47.	Laithangnu	Darlawn	Mizoram	Landrace
48.	Tai sanghar	Darlawn	Mizoram	Landrace
49.	Baimasa	Phuaibuang	Mizoram	Landrace
50.	Idaw	Tlungvel	Mizoram	Landrace
51.	Mangbuh	Chhingchhip	Mizoram	Landrace
52.	Buhpui	N Chaltlang	Mizoram	Landrace
53.	Fazu	Saichal	Mizoram	Landrace
54.	Jaiamenil	East Garo Hills	Meghalaya	Landrace
55.	Menilmibabaret	East Garo Hills	Meghalaya	Landrace
56.	TN1^a^	ICGEB	New Delhi	Improved
57.	BM71 ^a^	ABF, Hyderabad	Telangana	Improved
58.	IR71033–121-15B ^a^	ABF, Hyderabad	Telangana	Improved
59.	MO1 ^a^	ABF, Hyderabad	Telangana	Improved
60.	PTB33 ^a^	ABF, Hyderabad	Telangana	Improved
61.	CAUR1 ^b^	ICAR, Kolasib	Mizoram	Improved
62.	Gomati ^b^	ICAR, Kolasib	Mizoram	Improved
63.	RCM9 ^b^	ICAR, Kolasib	Mizoram	Improved
64.	RCM10 ^b^	ICAR, Kolasib	Mizoram	Improved
65.	RCM13 ^b^	ICAR, Kolasib	Mizoram	Improved

^a^represents*Indica* varieties, ^b^represents*Japonica* varieties. *ABF* Agri Biotech Foundation, *ICGEB* International Centre for Genetic Engineering and Biotechnology, *ICAR* Indian Council of Agricultural Research

### Estimation of Zn and Fe content

Dehusked rice seeds were crushed into a fine powder using mortar and pestle. The powdered sample (0.1 g) was placed in a 100 ml conical flask and 20 ml of Nitric acid (HNO_3_) was added to it. The mixture was kept on a hot plate till the fuming of nitrogen dioxide ceased. Another 20 ml of HNO_3_ was added and the samples were kept on the hot plate at a high temperature until the solution turned colourless. Then hydrogen peroxide (H_2_O_2_) was added to make the solution colourless. The mixture was heated until the solution was reduced to 3–5 ml. This extract was diluted to 20 ml with de-ionized water and then filtered through Whatman filter paper 1. The extract was then injected into Atomic Absorption Spectrophotometer (Shimadzu AA-7000, Japan) and the results were expressed in μg/g.

### Genomic DNA isolation and PCR amplification

Genomic DNA was isolated from 15-day old seedlings following Edwards et al. [43]. Single leaflet of 15-day old seedling was used for isolation of DNA. The leaflet was macerated using a micropestle in a 1.5 ml centrifuge tube. After maceration, 400 μl of extraction buffer (200 mM Tris HCl pH 7.5, 250 mMNaCl, 25 mM EDTA, 0.5% SDS) was added to the tube. The sample was then vortexed vigorously for 1 min and centrifuged at 13000 rpm for 5 min. Then, 300 μl of the supernatant was transferred to a fresh centrifuge tube and an equal volume of Isopropanol was added. The samples were kept at room temperature for 2 min and then were centrifuged at 13,000 rpm for 5 min. The resulting pellets were air dried at room temperature and dissolved in 100 μl TE (10 mM Tris, 1 mM EDTA) buffer.

Twenty-two simple sequence repeats (SSR) primers (Table 5) were used for amplification of genomic DNA. Amplification was performed in ABI Veriti 96 well Thermal cycler (ABI, USA) in 25 μl reaction containing 1X PCR buffer, 100 μM dNTP mixture, 3 mM MgCl_2_, 1 U Taq polymerase (Genie, India), 50 ng of each primer and 50 ng template DNA. The amplification conditions were set as, initial denaturation at 94 °C for 5 min, 35 cycles of denaturation at 94 °C for 30 s, annealing for 30 s, extension at 72 °C for 1 min followed by a final extension at 72 °C for 7 min. The amplified products were electrophoresed on 2.5% agarose gel and visualized by standard ethidium bromide staining [43, 44].

**Table 5:** Details of SSR primers used (http://gramene.org/markers/microsat/all-ssr.html)

Sl No	Primer name	Sequences (Forward primer/Reverse primer)	Chr.no.	Marker selection	T_a_ (°C)	Expected amplicon size (bp)
1.	RM1	Fp – 5′-GCGAAAACACAATGCAAAAA-3′ Rp – 5′-GCGTTGGTTGGACCTGAC-3’	1	Random	55	113
2.	RM154	Fp – 5’-ACCCTCTCCGCCTCGCCTCCTC-3′ Rp – 5′-CTCCTCCTCCTGCGACCGCTCC-3’	2	Random	61	183
3.	RM131	Fp – 5’-TCCTCCCTCCCTTCGCCCACTG-3′ Rp – 5′-CGATGTTCGCCATGGCGTCTCC-3’	4	Random	61	215
4.	RM135	Fp – 5’-CTCTGTCTCCTCCCCCGCGTCG-3′ Rp – 5′-TCAGCTTCTGGCCGGCCTCCTC-3’	3	Random	55	131
5.	RM153	Fp – 5’-GCCTCGAGCATCATCATCAG-3′ Rp – 5′-ATCAACCTGCACTTGCCTGG-3’	5	Random	55	201
6.	RM125	Fp – 5’-ATCAGCAGCCATGGCAGCGACC-3′ Rp – 5′-AGGGGATCATGTGCCGAAGGCC-3’	7	Random	55	127
7.	RM72	Fp – 5’-CCGGCGATAAAACAATGAG-3′ Rp – 5′-GCATCGGTACTAACTAAGGG-3’	8	Random	55	166
8.	RM171	Fp – 5’-CGATCCATTCCTGCTGCTCGCG-3′ Rp – 5′-CGCCCCCATGCATGAGAAGACG-3’	10	Random	55	328
9.	RM287	Fp – 5’-TTCCCTGTTAAGAGAGAAATC-3′ Rp – 5′-GTGTATTTGGTGAAAGCAAC-3’	11	Random	55	118
10.	RM302	Fp – 5’-TCATGTCATCTACCATCACAC-3′ Rp – 5′-ATGGAGAAGATGGAATACTTGC-3’	1	Trait-linked (drought)	55	156
11.	RM3825	Fp – 5’-AAAGCCCCCAAAAGCAGTAC-3′ Rp – 5′-GAGCTCCATCAGCCATTCAG-3’	1	Trait-linked (drought)	55	147
12.	RM246	Fp – 5’-GAGCTCCATCAGCCATTCAG-3′ Rp – 5′-CTGAGTGCTGCTGCGACT-3’	1	Trait-linked (drought)	55	116
13.	RM260	Fp – 5’-ACTCCACTATGACCCAGAG-3′ Rp – 5′-GAACAATCCCTTCTACGATCG-3’	12	Trait-linked (drought)	55	111
14.	RM525	Fp – 5’-GGCCCGTCCAAGAAATATTG-3′ Rp – 5′-CGGTGAGACAGAATCCTTACG-3’	2	Trait-linked (drought)	55	131
15.	RM219	Fp – 5’-CGTCGGATGATGTAAAGCCT-3′ Rp – 5′-CATATCGGCATTCGCCTG-3’	9	Trait-linked (drought)	55	202
16.	RM315	Fp – 5’-GAGGTACTTCCTCCGTTTCAC-3′ Rp – 5′-AGTCAGCTCACTGTGCAGTG-3’	1	Trait-linked (salt)	55	133
17.	RM223	Fp – 5’-GAGTGAGCTGGTGCTGAAAC-3′ Rp – 5′-GAAAGGCAAGTCTTGGCACTG-3’	8	Trait-linked (salt)	55	165
18.	RM8094	Fp – 5’-AAGTTTGTACACATCGTATACA-3′ Rp – 5′-CGCGACCAGTACTACTACTA-3’	1	Trait-linked (salt)	55	209
19.	RM493	Fp – 5’-TAGCTCCAACAGGATCGACC-3′ Rp – 5′-GTACGTAAACGCGGAAGGTG-3’	1	Trait-linked (salt)	55	211
20.	RM3412	Fp – 5’-AAAGCAGGTTTTCCTCCTCC-3′ Rp – 5′-CCCATGTGCAATGTGTCTTC-3’	1	Trait-linked (salt)	55	211
21.	RM443	Fp – 5’-GATGGTTTTCATCGGCTACG-3′ Rp – 5′-AGTCCCAGAATGTCGTTTCG-3’	1	Trait-linked (salt)	55	124
22.	RM169	Fp – 5’-TGGCTGGCTCCGTGGGTAGCTG-3′ Rp – 5′-TCCCGTTGCCGTTCATCCCTC-3’	5	Trait-linked (salt)	67	167

*Chr. no.* Chromosome number, *T*_*a*_ Annealing temperature

### Genetic data analysis

Bands were scored using Alpha View software (Alpha Imager, Protein Simple, USA). Total number of alleles, number of effective alleles, number of polymorphic loci, observed and expected heterozygosity, Nei’sgenetic diversity [45], Fst, and population-wise diversity were calculated using genetic analysis package POPGENE 1.31 [46]. Major allele frequency (MAF) and the polymorphism information content (PIC) were calculated using PowerMarker 3.25 [47]. Analysis of molecular variance (AMOVA) and principal co-ordinates analysis (PCoA) were performed in GenAlEx 6.5 [48]. The unweighted pair group method with an arithmetic mean (UPGMA) dendrogram was constructed using MEGA 6 [49] based on Nei’s genetic distance. The possible population structure was analyzed using STRUCTURE 2.3.4 [50]. The parameter was set as 100,000 for the length of burn-in period and Markov Chain Monte Carlo (MCMC) repeats after burn-in was set as 100,000. A possible number of subpopulations (K) was set from K = 1 to K = 10. Structure Harvester [51] was used to find the final K value. Then, the relationship among genetic diversity (gene diversity), Zn and Fe contents were measured using STATISTICA 5.0 (Statsoft Inc., USA, 1995).

## Supplementary information

**Additional file 1.** The genotype data of indigenous rice cultivars of NE India and improved varieties.

**Additional file 2.** Pairwise Population Matrix of Nei Genetic Identity.

**Additional file 3.** Pairwise Population Fst Values.

**Additional file 4.** Summary of analysis of molecular variance.

## Data Availability

All data supporting the conclusions of this article are included within the article and its Additional files 1, 2, 3 and 4.

## Abbreaviations

AMOVA: Analysis of molecular variance
Fst: Genetic differentiation
H_E_: Expected heterozygosity
HYV: High yielding variety
MAF: Major allele frequency
MCMC: Markov Chain Monte Carlo
na: Number of alleles
ne: Effective number of alleles
Nei: Nei’s genetic distance
PCoA: Principal co-ordinates analysis
PIC: Polymorphism information content
UPGMA: The unweighted pair group method with an arithmetic mean
